# Application of Micellar Mobile Phase for Quantification of Sulfonamides in Medicated Feeds by HPLC-DAD

**DOI:** 10.3390/molecules26133791

**Published:** 2021-06-22

**Authors:** Ewelina Patyra, Krzysztof Kwiatek

**Affiliations:** Department of Hygiene of Animal Feeding Stuffs, National Veterinary Research Institute, Partyzantów 57 Avenue, 24-100 Puławy, Poland; kwiatekk@piwet.pulawy.pl

**Keywords:** micellar liquid chromatography, sulfonamides, diode array detector, medicated feeds

## Abstract

Rapid chromatographic procedure for quantification of five sulfonamides in medicated feeds are proposed. Satisfactory separation of sulfonamides from medicated feeds was achieved using a Zorbax Eclipse XDB C18 column (4.6 × 150 mm, 5 µm particle size) with a micellar mobile phase consisting of 0.05 M sodium dodecyl sulphate, 0.02 M phosphate buffer, and 6% propan-**2**-ol (pH 3). UV quantitation was set at 260 nm. The proposed procedure allows the determination of sulfaguanidine, sulfadiazine, sulfamerazine, sulfamethazine, and sulfamethoxazole in medicated feeds for pigs and poultry. Application of the proposed method to the analysis of five pharmaceuticals gave recoveries between 72.7% to 94.7% and coefficients of variations for repeatability and reproducibility between 2.9% to 9.8% respectively, in the range of 200 to 2000 mg/kg sulfonamides in feeds. Limit of detection and limit of quantification were 32.7–56.3 and 54.8–98.4 mg/kg, respectively, depending on the analyte. The proposed procedure for the quantification of sulfonamides is simple, rapid, sensitive, free from interferences and suitable for the routine control of feeds. In the world literature, we did not find the described method of quantitative determination of sulfonamides in medicated feeds with the use of micellar liquid chromatography.

## 1. Introduction

Antibiotics are natural or synthetic origin drugs that have the capacity to kill or inhibit the growth of microorganisms [1]. Over the last decades, livestock production has increased notably, mainly due to intensive farming. Veterinary drugs have been extensively used in animal husbandry, both for prophylactic and therapeutic purposes [2]. One group commonly used as antibacterial drugs in both human and veterinary medicine are sulfonamides (SAs). Sulfonamides are synthetic antimicrobial compounds that are widely used to treat respiratory, gastrointestinal, and urinary tract infections caused by microorganisms resistant to other antibiotics [3]. According to the ninth European Surveillance of Veterinary Antimicrobial Consumption (ESVAC) report, which compiled sales of veterinary antimicrobial agents in 31 European countries during 2017, the largest quantities of antimicrobials sold were tetracyclines (30.4%), penicillins (26.9%), and sulfonamides (9.2%). Overall, these three classes accounted for 66.5% of total sales in the 31 countries [4]. Sulfonamides are in the one group antibacterial substances which can be administered to livestock as a medicated feeds. According to the Regulation (EU) 2019/4 of the European Parliament and of the Council of 11 December 2018 medicated feed is a homogeneous mixture of feed and veterinary medicinal products. ‘Medicated feed’ means a feed, which is ready to be directly fed to animals without further processing, consisting of a homogenous mixture of one or more veterinary medicinal products or intermediate products with feed materials or compound feed [5]. To ensure the protection of animal health in the European Union countries control of medicated feed is carried out in terms of the content of antibiotics in the feed and its homogeneity. Therefore, it is necessary to develop and implement appropriate analytical methods to control the content of veterinary medicines in feeds.

In scientific literature many methods have been reported for analysis of sul-fonamides in biological matrices [6,7,8,9,10,11]. A considerable number of methods have been developed for determination of sulfonamides in tissues, eggs, honey, milk and biological fluids [6,7,8,9,10,11]. However, only a few publications describe analytical methods that allow the quantification of sulfonamides in medicated feed. The published methods for determination of sulfonamides in medicated feed used the techniques of enzyme-linked immunosorbent assay (ELISA) [12], microbiological assay [13], or liquid chromatography with photodiode detector or mass spectrometry [14,15]. The lack of a higher number of methods could be caused by the fact that feed samples are very complex and have variable matrixes and some feed components are easily coextracted with the analytes, which may disturb the analysis. Therefore, we have proposed a new solution for the analysis of sulfonamides in medicated feeds using micellar liquid chromatography (MLC) in order to minimize the use of organic sovents for chromatographic analysis.

In recent years, a technique known as micellar liquid chromatography (MLC) has been used as an alternative method to conventional liquid chromatography. Micellar liquid chromatography is one of the modes of reversed phase liquid chromatography (RPLC) in which the mobile phases are aqueous solutios of a surfactant at a concentration above the critical micelle concentration (CMC) [16]. Over the past 15 years, the popularity of MLC has grown rapidly. The idea of using micellar solutions as mobile phases in RPLC is very attractive owing to the lower cost and toxicity and the reduced environmental impact. In addition, micellar phases are less flammable, non-toxic, and biodegradable [17,18]. The MLC technique is based on the use of a surfactant. The choice of surfactant is often limited to the following surfactants: anionic sodium dodecyl sulfate (SDS), cationic ce-tyltrimethylammonium bromide (CTAB), and nonionic polyoxyethylene lauryl ether (Brij-35) [19]. Most MLC methods use hybrid mobile phases consisted of aqueous so-lutions of a surfactant above its CMC and a small portion of organic modifier (mostly 3‒15%, *v*/*v*). Micellar mobile phases are safer for both the operator and environment [20].

The aim of this work was to optimize the isocratic mobile phase based on MLC along with using SDS as the modifier agent for simultaneous isolation and quantification of sulfonamides: sulfaguanidine (SGD), sulfadiazine (SDZ), sulfamerazine (SMR), sulfametazine (SMZ), and sulfamthoxazole (SMX) in medicated feeds. To our knowledge, the stability-indicating green reverse phase HPLC (RP-HPLC) method using environmentally benign eluents (propan-**2**-ol) has not been reported in the literature for the analysis of sulfonamides in medicated feed. Therefore, the aim of the presented study was to optimize, develop and validate a simple, cost effective, rapid, facile, selective, precise, reproducible, accurate, robust, and stability-indicating green MLC method coupled with UV detection for the rapid analysis of sulfonamides in medicated feeds.

## 2. Results and Discussion

### 2.1. Optimization Strategy and Mobile Phase Selection

Many authors have been interested in the applications of MLC for separation different active compounds such as antibiotics, vitamines, alkaloids, antidepressants, steroids, diuretics, and cardiovascular drugs [21,22,23,24,25,26,27,28,29,30,31,32,33]. The main advantages of this method include unique selctivity related to the interaction between the micelle and the chromatographed substances is a possibilty of separation of the ionic and nonionic substanceson the same packing of the chromatography column, low cost of the mobile phase, nontoxicity, and nonflammability of the mobile phase and possibility of determination of the biologically active substances by a direct sample injection technique without prelimary separation of the analyte [28].

Szymański [28,34,35], Garcia et al. [36] and Raviolo et al. [37] used micellar mobile phase for detection and separation sulfonamides in different kinds of matrices e.g., in milk, blood, honey, meat and pharmaceuticals formulations. Sulfonamides are of amphoteric character, the main parameter influencing the retention and permitting optimization of the separation system is the pH of the mobile phase. Sulfonamides can occur in three forms: undisociated molecules, anions or cations depending on the pH values. Therefore, the pH value of the mobile phase is a very important factor for these compounds. Some physicochemical properties of the investigated drugs are given in Table 1.

The proposed method permits the quantitation of SGD, SDZ, SMR, SMZ, and SMX in medicated feeds. To achieve the best chromatographic conditions, the mobile phase composition was optimized to provide sufficient selectivity and sensitivity in short analysis time.

In this work extensive experimental studies were carried out to select the most efficient parameters for the analysis. The final experimental conditions were chosen after testing the type of stationary phase, composition of the mobile phases and detection wavelength. The parameters to be optimized in this work were the concentrations of the main components of the hybrid mobile phase (pH, SDS, propan-**2**-ol, buffer solutions) and the detection conditions. The study was performed using a standard solution containing 0.1 µg/mL of sulfonamides.

#### 2.1.1. Choice of Appropriate Detection Wavelength

In the Table 1 are presented maximum wavelenght for all analysing sulfonamides. We checked UV wavelengths between 220 and 280 nm and finally we selected the wavelength of 260 nm as the best for all five sulfonamides.

#### 2.1.2. Choice of Column

For selection of a suitable stationary phase for separation of sulfonamides, two chromatographic columns were investigated including: Zorbax Eclipse XDB C8 and Zorbax Eclipse XDB C18 (4.6 mm 150 mm, 5 µm) both from Agilent Technologies (Santa Clara, CA, USA). The use of the Zorbax Eclipse XDB C8 column did not give satisfactory results. The analyzed sulfonamides were not well separated on this chromatografic column. The next step, employing a Zorbax Eclipse XDB C18 column, was found to be the most suitable, giving symmetrical well resolved peaks for all five sulfonamides within a short analysis time.

#### 2.1.3. Selection of SDS Concentration

In order to find the best composition of the mobile phase that allows the simultaneous analysis of the five sulfonamides considered in this study each of them was injected into the mobile phase containing 0.02 M phosphate buffer and SDS in five different concentrations: 0.03, 0.04, 0.05, 0.06, and 0.07 M at pH 3. The retention of the sulfonamides were excessive when eluted with a purely micellar SDS mobile phase (without organic modifier), with the retention times larger than 40 min. The study showed that sulfonamides bind to micelles, as the retention and the efficiency decreased at increasing values of SDS in the mobile phase. This was probably due to the electrostatic attraction between the analytes and the micelles [39,40]. Table 2 shows the effect of SDS concentration on the separation of the analyzed SAs, peak shape, time of analysis and column pressure. As a result of the experiment, the optimal concentration of SDS was found to be 0.05 M. Further experiments were carried out with the use of 0.02 M phosphate buffer and 0.05 M SDS at pH = 3.

#### 2.1.4. pH Selection

Sulfonamides can have various substituents (R1 and R2), both on the amino and sulfonamide groups, and depending on their kind, they can be acidic and/or basic, or neutral. The basic character is given to them by the unsubstituted amino group on the aromatic ring, while the acidic character is given by the sulfonamide group. Since the nature of analytes, we tested acidic mobile phase with different pH and two different buffer solution: oxalic buffer (pH 2–5) and phosphate buffer (pH 2–5) were tested. The best peak shapes in the chromatogram were obtained using phosphate buffer. The analyzed pH of the phosphate buffer in the range from 2 to 5 showed that the best results were obtained for pH 3, therefore, for further analyzes, 0.02 M phosphate buffer at pH 3 was used.

#### 2.1.5. Use of an Organic Modifier

Most analytical procedures in micellar liquid chromatography require the addition of an organic solvent, which increases the peak efficiency. Usually it is a short-chain alcohol such as propan-**2**-ol, butanol or pentanol. When a surfactant (e.g., SDS) and a small addition of alcohol (3–15%) are used in the mobile phase, scientists call this combination hybrid micellar mobile phase [20]. Introduction of an organic modifier into a micellar mobile phase has beneficial effect on the efficiency of chromatographic distribution [35]. The addition of a small amount of organic solvent into a micellar mobile phase reduces the amount of the surfactant adsorber on the surface of the stationary phase which improves the column efficiency [33,35]. In this work, we tested butanol and propan-**2**-ol as organic modifier. The concentration of butanol and propan-**2**-ol was tested in the range of 4% to 10%. Propan-**2**-ol gave better efficiencies than butanol, better separation the five sulfonamides, lower pressure on the chromatographic column and better analysis times below 20 min. All experiments are shown in Table 3. A concentration of 6% *v*/*v* propan-**2**-ol was chosen as the optimal concentration, where it provided a good combination of peak symmetry, sharpness, resolution factor, and short analysis time.

**Figure 1:**
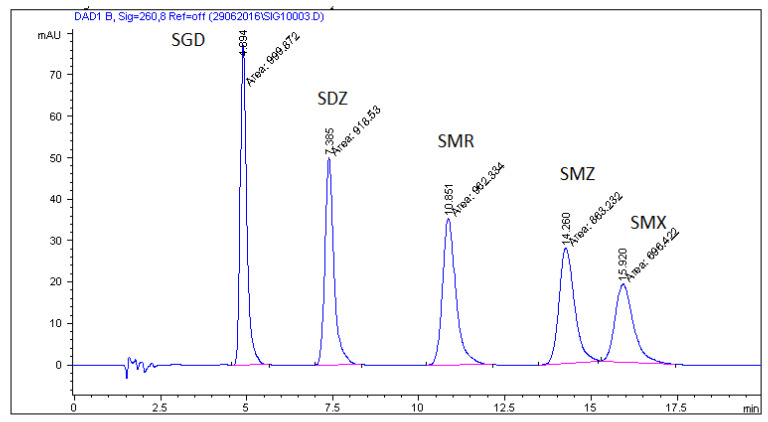
Chromatogram of standard solution of sulfonamides in the MLC system (0.05 M SDS/0.02 M phosphate buffer containing 6% propan-**2**-ol, pH = 3 at a flow rate of 0.6 mL/min).

After this experimental study, when maximum resolution–minimum analysis time criteria were applied, the mobile phase selected as being optimal was 0.05 M SDS–0.02 M Na_2_HPO_4_ and 6% of propan-**2**-ol at pH 3 with UV-detection at 260 nm. Figure 1 illustrates a representative chromatogram for SAs in the pure drug substance under the optimum chromatographic conditions.

### 2.2. Sample Preparation

**Figure 2:**
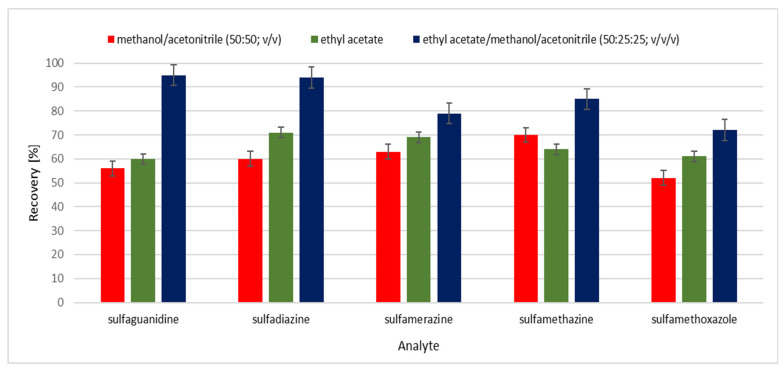
Effect of the extraction solution for recovery of the analyzed compounds (*n* = 3).

**Figure 3:**
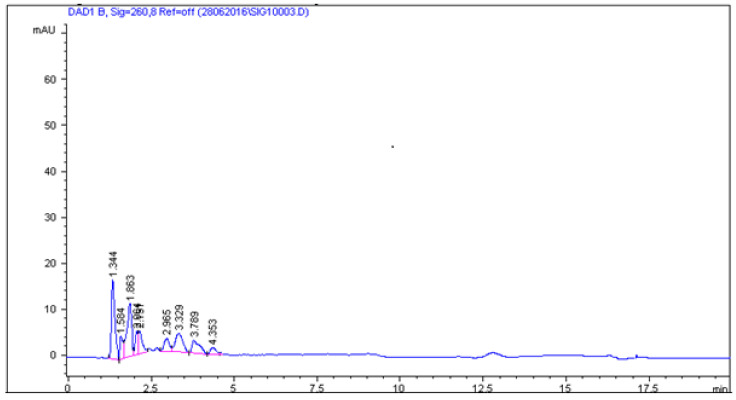
Blank feed sample.

**Figure 4:**
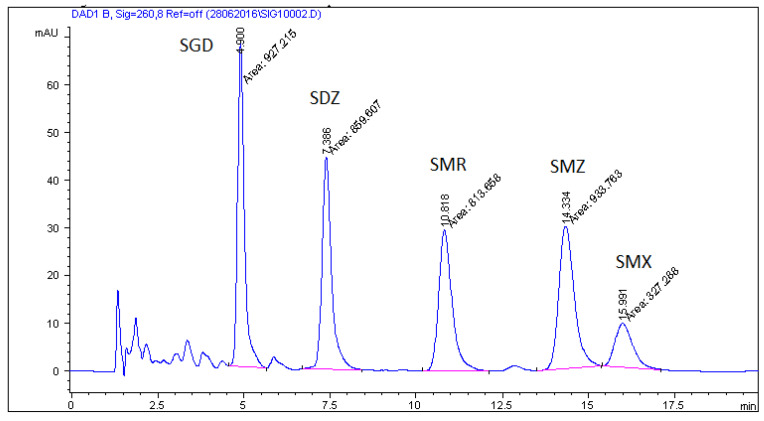
Feed sample spiked with all analyzed sulfonamides at a concentration 200 mg/kg (SGD—sulfaguanidine; SDZ—sulfadiazine; SMR—sulfamerazine; SMZ—sulfamethazine; SMX—sulfamethoxazole).

**Figure 5:**
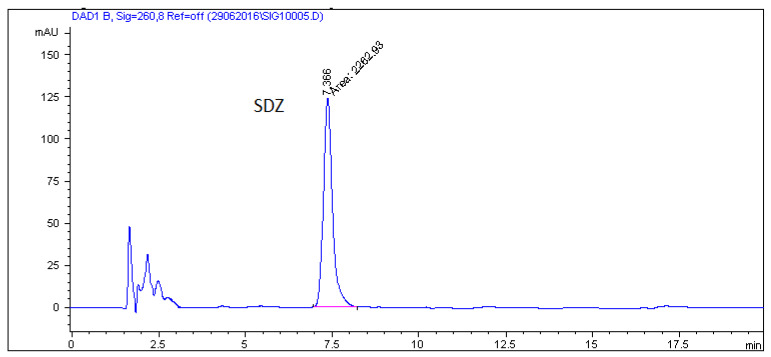
Real medicated feed sample with sulfadiazine at a concentration 450 mg/kg.

Various methods, including extraction protocols, can be found in the literature for the analysis of sulfonamides in food matrices such as meat, milk, eggs, honey [35,36,37,41] and currently is some publications where are described method analysis these compounds in non-target feed [3,41], but extraction and analysis methods of this antibacterial substances in medicated feeds are still rare. Stringham et al. [42] extracted sulfonamides from feed and feed premixes by shaking with 0.15 N HCl in 25% methanol. Next, extract was diluted, clarified, and chromatographed. In 2011 Przeniosło-Siwczyńska et al. [15] published procedure for detection and quantification sulfaguanidine in medicated feed where they use water and acidified methanol mixture and after extraction, extract was diluted 1000 times and injected onto the LC-MS (APCI) instrument. Pietroń and co-workers [14] for the extraction sulfaguanidine, sulfadiazine, sulfamethazine, sulfamethizole, and sulfamethoxazole used mixture consisting of methanol and acetonitrile (50:50 *v*/*v*) and HPLC-DAD analysis. In our work, in the first stage, we used the extraction method proposed by Pietroń et al. based on the use of a mixture of methanol and acetonitrile, but the recoveries for the five analyzed SAs were low, from 50% to 70%. Therefore, we have made an attempt to optimize the extraction method in order to obtain the highest possible recovery for all five tested sulfonamides. We tested extraction solutions consisting of methanol and acetonitrile, ethyl acetate, and a combination of methanol, acetonitrile and ethyl acetate. Results are shown in Figure 2. In our work the best recoveries for five sulfonamides were obtained for a mixture of ethyl acetate/methanol/acetonitrile in proportion 50:25:25, *v*/*v*/*v*. After extraction one milliliter of extract was evaporated under nitrogen stream and next resuspended in phosphate buffer pH = 7. Use of ethyl acetate/methanol/acetonitrile mixture allows obtaining high recoveries of SAs without additional purification step. Micelles in the mobile phase strongly interact with proteins, fats and other macromolecules. Therefore, they are easily solubilized in a micellar solution, and then the suspension obtained from the solid/liquid extraction of animal feed can be directly injected, after centrifugation and filtration, without risk of damaging the column [39]. Combination of extraction method and the use of micellar mobile phase allows obtaining clear chromatographic images without any interferences from feed matrices. Blank feed sample and feed samples spiked with all analysed sulfonamides which were analyzed with the mobile phase consisting of 0.02 M phosphate buffer-0.05 M SDS-6% propan-**2**-ol (pH 3) are shown in Figure 3 and Figure 4. In Figure 5 are shown real medicated feed sample from feed mills with sulfadiazine at a concentration 450 mg/kg.

### 2.3. Validation of the Method

The developed analytical procedure was in-house validated following the guidelines of the EU Commission Decision 2002/657/EC, in order to check its reliability in the considered range [43]. The studied validation parameters were: selectivity, linearity, calibration range, intra- and interday accuracy and precision. The limits of detection (LOD) and limits of quantification (LOQ) were determined by the ICH Harmonized Tripartite Guideline, as the EU Commission Decision does not mention them [44].

Due to the lack of certified reference material for all analytes investigated, the method was validated using recovery of known amounts of analytes spiked into blank samples. The linearity of the HPLC-DAD response was proved with working standard solutions at five concentration levels (200, 500, 1000, 1500, and 2000 mg/kg). Recovery and precision assay of the proposed method were evaluated using spiked feed samples at the three different levels (Table 4). The relative SGD, SDZ, SMR, SMT, and SMX recoveries for micellar liquid chromatography with diode array detection method ranged from 72.7% to 94.7% for all analyzed compounds. The repeatability and within-laboratory reproducibility for five antibacterial substances were lower than 9% for repeatability and 10% for within-laboratory reproducibility. LOD and LOQ values for SGD, SDZ, SMR, SMZ, and SMX in medicated feed ranged from 32.7 mg/kg to 56.3 mg/kg and 54.8 mg/kg to 98.4 mg/kg, respectively. Pietroń et al. obtained lower LOD and LOQ values for sulfonamides ranging from 2 to 10 mg/kg and 5.2 to 30.2 mg/kg, respectively, when using normal RP-HPLC [15]. The use of normal RP-HPLC allows for lower LOD and LOQ values compared to micellar liquid chromatography, but the analysis time of a single sample is longer andthe obtained chromatogram of the feed sample shows significantly more interference from the feed matrix compared to the presented micellar mobile phase.

The specificity was studied by analyzing the blank samples of feed for poultry and pigs. In all cases, several peaks were detected to 4 min, corresponding to the matrix endogenous compounds. No peaks were observed near the retention times of the analytes. Validation parameters are shown in Table 4.

## 3. Material and Methods

### 3.1. Standards, Chemicals, and Reagents

SDZ, SGD, SMR, SMT, and SMX standards and SDS were from Sigma Aldrich (St. Louis, MO, USA). Analytical grade solvents, acetonitrile, methanol and propan-**2**-ol were from J.T. Baker (St. Louis, MO, USA). Ethyl acetate and disodium hydrogen phosphate were purchased from POCH (Gliwice, Poland). High purity water with a resistivity of 18.2 MΩ cm was obtained from a Milli-Q water system (Millipore, Bedford, MA, USA).

### 3.2. Preparation of Standard and Mobile Phase Solutions

The micellar mobile phases were prepared by weighing the appropriate amounts of SDS and disodium hydrogen phosphate. These reagents were dissolved in ultrapure water. The pH of the micellar eluent was adjusted to 3 before the addition of propan-**2**-ol for mobile phase.

Standard stock solutions of individual sulfonamides (10 mg/mL) were prepared in methanol for sulfaguanidine, sulfamethazine, sulfamerazine, and sulfamethoxazole. A sulfadiazine (SDZ) standard was prepared by dissolving in acetonitrile. They were stored in a freezer at −20 °C and found to be stable for six months.

### 3.3. Calibration Curves

A series of working standard solutions were prepared at the concentrations of 0.04, 0.1, 0.2, 0.3, and 0.4 mg/mL of SGD, SMZ, SMR, SMX, and SDZ in 0.02 M phosphate buffer, pH 7. These solutions were analyzed by HPLC-DAD and calibration curve was plotted.

### 3.4. Feed Samples

Samples were collected from feed mills and farms in Poland. Samples of complete feed for poultry and pigs were consisting of cereals as corn, wheat, soybeans, wheat bran, sunflower meal, feed chalk, feed phosphate, and dietary additives (vitamins and micro- and macro-elements) zootechnical additives and technological additives. Prior to use, the absence of veterinary drugs in blank samples were confirmed by HPLC-DAD analysis and by applying the developed method.

### 3.5. Extraction and Clean-Up

A feed sample of 2.00 ± 0.01 g, previously grinded, was weighed into a 50 mL polypropylene centrifuge tube. To prepared feed samples 20 mL of ethyl acetate/acetonitrile/methanol (5; 2.5; 2.5, *v*/*v*/*v*) mixture was added. The samples were shaken for 30 min on a horizontal shaker and centrifuged for 20 min, at 4000× *g*. One mL of the extract was dried under a gentle stream of nitrogen. The residue was reconstituted in 1 mL of a phosphate buffer pH 7 and filtered through 0.45 μm syringe filters and injected into the chromatographic system.

### 3.6. Chromatographic Conditions

The HP 1100 Series separation modules Agilent Technologies (Santa Clara, CA, USA) was used for the analysis. The chromatographic separation was accomplished with isocratic elution on Zorbax Eclipse XDB column C18, 150 × 4.6 mm, 5 μm (Agilent Technologies, Santa Clara, CA, USA), using a MLC mobile phase consisting of 0.05 M sodium dodecyl sulphate/0.02 M phosphate buffer (pH = 3) and 6% propan-**2**-ol mixture prepared in one glass bottle. The flow rate was 0.6 mL/min and the column thermostat was set at 30 °C. The injection volume was 10 μL. The UV detection was monitored at 260 nm.

### 3.7. Validation Studies

The method was in-house validation with the criteria specified by European Commission Decision 2002/657/EC and ICH guideline [43,44]. The linearity of the method was evaluated using standard solutions calibration curves with five concentration levels in triplicate in the range 0.004–0.4 mg/mL. To verify the absence of interfering endogenous compounds around the retention time of analytes 10 different blank feed samples for poultry and pigs were analyzed. LOD and LOQ for all sulfonamides were determined with the use signal-to-noise (S/N) of 3 and 10, respectively. To determine recovery six blank feed samples spiking with known amounts of the sulfonamides at three different concentration (200, 1000, and 2000 mg/kg, six replicates for each level). Recovery values were calculated by comparing the concentration obtained from the feed samples with the added amounts. Repeatability was assessed by comparing the results of six replicates prepared the same day at three different concentrations (200, 1000, and 2000 mg/kg). The procedure was repeated to determine within-laboratory reproducibility by comparing results from samples prepared and analysed on three different days. Standard deviations (SD) and coefficients of variation (CV, %) were calculated for each level.

## 4. Conclusions

The rapid and satisfactory extraction procedure for five sulfonamides from animal medicated feed has been demonstrated. We proposed new MLC method for sulfonamides based on the use of conventional C18 column. Moreover, the use of micellar liquid chromatography technique is advantageous because it protects analysts from the exposure to volatile organic solvents during chromatographic analysis Micellar liquid chromatography has proved to be a suitable, simple, and rapid technique to analyse sulfonamides in medicated feed samples. In addition, the selected mobile phase is cheaper and less toxic than those used in conventional RPLC. Moreover, the use of micellar liquid chromatography technique is advantageous because it protects analysts from the exposure to volatile organic solvents during chromatographic analysis. Described chromatographic procedure is useful for routine quantification analysis of the sulfonamides in medicated feeds for pigs and poultry.

## Figures and Tables

**Table 1:**
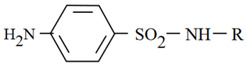
Structure and some physicochemical properties of the investigated sulfonamides [34,38].

Sulfonamide	R	pK_a1_	pK_a2_	logP_ow_	λ_max_
Sulfaguanidine	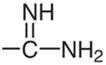	2.8	12.1	−1.22	260
Sulfadiazine	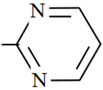	2.0	6.4	−0.18	218
Sulfamerazine	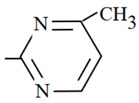	2.2	7.0	0.06	261
Sulfamethazine	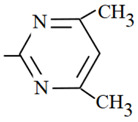	2.1	7.4	0.32	268
sulfamethoxazole	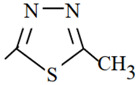	1.7	5.6	0.91	270

**Table 2:** Optimization of SDS concentration in the mobile phase.

Parameters	SDS Concentration (mol/dm^3^)				
	0.03	0.04	0.05	0.06	0.07
Peak separation	No separation	No separation	Good separation	Good separation	Good separation
Column pressure (bar)	<60	<70	>80	90–100	>100
Peak shape	not Gaussian shape	not Gaussian shape	Gaussian shape	Gaussian shape	Gaussian shape
Time of analysis (min)	>40	>30	<25	<20	<20

**Table 3:** Optimization of propan-**2**-ol concentration of the mobile phase.

Parameters	Propan-2-ol Concentration (%)						
	4	5	6	7	8	9	10
Peak separation	No separation	No separation	Good separation	Good separation	No separation	No separation	No separation
Column pressure (bar)	>70	>70	<70	<70	60	50–60	50–60
Peak shape	Not Gaussian shape	Not Gaussian shape	Gaussian shape	Gaussian shape	Gaussian shape	Gaussian shape	Gaussian shape
Time of analysis (min)	>30	>30	<20	<20	<20	<15	<15

**Table 4:** Validation parameters of optimized MLC-DAD method.

Analyte	Linearity R^2^	Linearity Range (mg/kg)	Intra-Day, CV% (mg/kg)			Inter-Day, CV% (mg/kg)			Recovery (%) (mean ± S.D.)	LOD (mg/kg)	LOQ (mg/kg)	U (%)
			200	1000	2000	200	1000	2000				
SGD	0.9976	0.004–0.40	5.7	2.9	3.3	7.2	5.5	4.3	94.7 ± 7.2	33.1	54.8	10.4
SDZ	0.9954	0.004–0.40	6.1	3.3	5.0	8.0	4.3	6.7	94.4 ± 8.7	38.9	70.4	16.0
SMR	0.9997	0.004–0.40	8.8	2.9	3.1	9.3	9.0	5.9	78.9 ± 10.7	32.7	63.2	17.1
SMZ	0.9991	0.004–0.40	7.4	6.0	5.6	6.9	7.6	7.6	85.3 ± 2.1	42.3	74.7	14.5
SXZ	0.9978	0.004–0.40	6.8	8.6	7.1	9.2	8.8	9.8	72.7 ± 8.4	56.3	98.4	18.0

## Data Availability

The data presented in this study are available in the article.

## Sample Availability

Samples of the compounds are not available from the authors.
